# Genomic characterization of non-O1, non-O139 *Vibrio cholerae* causing rare clinical manifestation

**DOI:** 10.1186/s12879-025-12191-9

**Published:** 2025-12-09

**Authors:** Judit Henczkó, Márta Vargha, András Kállai, Katalin Kristóf, Ákos Tóth, Szilárd Tóth, Bernadett Pályi, Bernadett Khayer, Eszter Róka, Bereniké Novák, Tünde Mag, Eszter Mezei, Zsuzsanna Molnár, Zoltán Kis

**Affiliations:** 1 National Biosafety Laboratory, National Center for Public Health and Pharmacy, Budapest, Hungary; 2 School of PhD Studies, Semmelweis University, Budapest, Hungary; 3 Division of Public Health Laboratory and Methodology, Department for Environmental Health Laboratory, National Center for Public Health and Pharmacy, Budapest, Hungary; 4 Department of Anesthesiology and Intensive Therapy, Semmelweis University, Budapest, Hungary; 5 Department of Laboratory Medicine, Semmelweis University, Budapest, Hungary; 6 Division of Reference Laboratories for Microbiology, National Center for Public Health and Pharmacy, Budapest, Hungary; 7 Communicable Diseases and Immunization Unit, National Center for Public Health and Pharmacy, Budapest, Hungary; 8 Institute of Medical Microbiology, Faculty of Medicine, Semmelweis University, Budapest, Hungary

**Keywords:** Non-O1, Non-O139 *Vibrio cholerae*, Pneumonia, WGS, cgMLST

## Abstract

**Background:**

Non-O1, non-O139 *Vibrio cholerae* is an uncommon cause of pneumonia, particularly following freshwater exposure. Non-O1, non-O139 *Vibrio cholerae* was identified from bronchoalveolar lavage through culture and quantitative polymerase chain reaction (qPCR) in Hungary. During an epidemiological investigation, the source of infection was traced to a designated bathing site at a lake in Central Hungary, where non-O1,non-O139 *Vibrio cholerae* was isolated from surface water.

**Methods:**

We conducted whole-genome sequencing and comparative genomic analysis on a clinical isolate (*N* = 1) and three phenotypically distinct environmental isolates (*N* = 3). In addition, we reviewed the available literature on pulmonary infections associated with *Vibrio cholerae*.

**Results:**

Core genome multilocus sequence typing (cgMLST) revealed that the clinical and environmental isolates clustered together with zero allelic differences. Multilocus sequence typing (MLST) identified a new sequence type (ST1605), representing a novel combination of known allele variants. In silico analysis of antibiotic resistance genes identified the presence of *bla*CARB-7. Both the clinical and environmental isolates exhibited identical virulence gene profiles, reinforcing the hypothesis that the infection was acquired from a local water source.

**Conclusions:**

This study represents the first investigation of a primary pulmonary *Vibrio cholerae* infection reported in Europe following a near-drowning event. While *Vibrio vulnificus* and *Vibrio metschnikovii* have been implicated in similar pneumonia cases, the precise virulence mechanisms of these species remain poorly understood. Although non-O1, non-O139 *Vibrio cholerae* infections associated with recreational water exposure are rare in Hungary (1–2 cases per year), this study underscores the importance of ongoing surveillance for the detection of potential outbreaks and to inform public health responses.

**Supplementary Information:**

The online version contains supplementary material available at 10.1186/s12879-025-12191-9.

## Background

 Non-toxigenic *Vibrio cholerae* (NTVC) occurs naturally in both marine and freshwater environments [1]. In recent decades, NTVC has been detected in various lakes, rivers, and seawaters, including the North Sea, the Baltic Sea, and the Mediterranean Sea. Humans can be infected through the consumption of contaminated fish or seafood, or through direct water exposure. The incidence of human NTVC cases is uncertain, but the number of reports on their prevalence in water environments and their human infections have been increasing across Europe in recent years [1–5]. In addition, a recent large-scale European study analyzing 63 clinical and 24 environmental isolates provides important background information supporting the relevance of NTVC in Europe [5]. There is a strong association between NTVC incidence and elevated water temperature, increased salinity or dissolved oxygen levels, and it is well known historically that *Vibrio* species have the fastest growth rates among bacteria, responding almost immediately to favorable environmental conditions [6]. NTVC can be also isolated from various fish, mollusks, shrimps, water birds and mammals [7]. NTVC are often found in the environment in a viable but non-culturable form (VBNC) and are frequently found in biofilms [8, 9]. Although more than 200 *Vibrio cholerae* serogroups are currently known, the cholera toxin gene is usually carried by O1 and O139. However, other serogroups, such as O75 or O141 are sometimes toxigenic and responsible for smaller epidemics [10]. Non-O1, non-O139 *Vibrio cholerae*, has also been implicated as etiologic agents of asymptomatic to severe human diseases, including gastroenteritis and extraintestinal infections, such as sinusitis, otitis, sepsis, peritonitis, conjunctivitis, wound infections and soft tissue infections [2, 11–18]. Infections are usually self-limiting, but underlying conditions (e.g. immunosuppressed patients) may increase the risk of severe infection. The healthcare system only recognizes laboratory-confirmed cases, which are the tip of the iceberg [1]. Empiric antibiotic therapy is recommended as a first- line treatment for extraintestinal infections and for the two most commonly identified types of infection [19–21].

Non-toxigenic serogroups are invisible to public health systems in most countries, posing a potential public health risk. Passive surveillance has been available in Hungary since the early 1950s; however, all *Vibrio cholerae* isolates should be sent to a reference laboratory for species confirmation and cholera toxin detection. In this case report, we present details of this atypical infection and the genomic features of the isolates. To our knowledge, this is the first case of primary pneumonia in Europe caused by non-O1 and non-O139 *Vibrio cholerae*.

## Methods

### Clinical sample

A previously healthy 21-year-old male patient was admitted to the Intensive Care Unit (ICU) of the Department of Anesthesiology and Intensive Therapy, Semmelweis University, Budapest, in critical condition after a near-drowning event and two minutes of resuscitation. The primary diagnosis was adult respiratory distress syndrome (ARDS), characterized by bilateral infiltrates on chest X-ray and a low PaO2/FiO2 ratio (the lowest value recorded was 109 within the first 24 h, despite the application of 10 cmH₂O positive end-expiratory pressure (PEEP)). A bronchoalveolar lavage (BAL) sample taken shortly after admission revealed no significant bacterial colony count.

After an initial two-day period of transient improvement, the condition of the patient deteriorated, characterized by the onset of fever and impaired gas exchange. In response to the worsening of ventilatory status, prone positioning and airway pressure release ventilation (APRV) were initiated. Markedly elevated inflammatory markers (peak values on day 4 of treatment: procalcitonin 230 µg/L and CRP 368 mg/L), together with the development of purulent tracheal secretions, raised suspicion of aspiration pneumonia, supported by chest X-ray and computed tomography findings. The CT scan additionally revealed a 2-cm pulmonary abscess.

*Vibrio* species, identified with a low confidence score using matrix-assisted laser desorption ionization time-of-flight mass spectrometry (MALDI-TOF MS, Biotyper^®^, database v.1.), were cultured in significant colony counts from a second BAL sample. In line with the national guidelines, the laboratory sent the clinical isolate to the National Biosafety Laboratory, National Center for Public Health and Pharmacy, for further confirmation, where the presence of non-O1, non-O139 *Vibrio cholerae* was confirmed using classical and molecular methods (see below).

Following a complex intensive care approach, which included prone positioning with APRV ventilation, combined high-dose vasopressor and inotropic therapy, and six days of continuous renal replacement therapy, antibiotic therapy was administered based on consultation with infectious disease specialists. Treatment was initiated with empiric amoxicillin/clavulanic acid, which was subsequently escalated to meropenem and cefazolin based on culture results, leading to stabilization of the patient. This antibiotic therapy was maintained until day 20 of treatment; the antibiotic therapy was discontinued due to a decrease in inflammatory markers and the absence of infectious clinical symptoms. Given that previous case reports occasionally documented fungal infections following near-drowning in freshwater, empiric antifungal therapy with amphotericin B was initiated. This treatment was subsequently discontinued based on culture results and consultation with infectious disease specialists.

After a prolonged critical condition complicated by a lung abscess, the patient spent 27 days in the ICU and 34 days in the hospital. After the accident, a standard epidemiological investigation was performed and identified the lake in Central Hungary with the exact location where the near-drowning event occurred. The site of exposure was a designated European Union bathing site. The patient fully recovered after the therapy.

### Bacterial isolates and antibiotic susceptibility

The clinical isolate was plated on Thiosulfate-Citrate-Bile-Sucrose agar plates, TCBS (Merck, Darmstadt, Germany) and Vibrio ChromoSelect Agar (Sigma, Germany) and incubated for 24 h at 37 °C under ambient air. Additionally, sheep blood agar (SBA) was inoculated and incubated at 37˚C under ambient air for 24 h. For rapid identification of the main toxigenic serogroups, in-house slide agglutination was performed using rabbit anti-*V. cholerae* O1 and O139 sera (National Center for Public Health and Pharmacy, Budapest) from a 24-hour log-phase growth culture. We performed basic biochemical tests, including oxidase, catalase and indole tests.

An accredited laboratory collected a water sample at the exact location one week after the exposure in August 2023 using a sampling rod in a sterile 500 mL glass bottle, from a water depth of 30 cm. Water temperature, electrical conductivity, pH and oxygen content were measured directly during sampling using a portable device. Aliquots of the water sample from 1 mL to 100 mL were filtered through 0.45 μm pore-size mixed cellulose ester membrane filters (Ø 47 mm; Merck, Darmstadt, Germany), which were placed onto TCBS agar (Merck, Darmstadt, Germany), and Vibrio ChromoSelect Agar (Sigma, Germany) plates in parallel and incubated at 37 °C under ambient air for 24 h. Typical bacterial colonies on the membrane filters were enumerated according to the ISO 8199:2018 standard [22] and expressed as colony-forming units (CFU) per 100 mL. The isolates were identified using MALDI -TOF MS Biotyper Syrius One, (database v. 7.) Antimicrobial susceptibility testing was performed according to the EUCAST disk diffusion technical guideline [21] using cefotaxime (5 µg) ceftazidime (10 µg), doxycycline (30 µg), pefloxacin (5 µg), meropenem (10 µg), erythromycin (15 µg), trimethoprim-sulfamethoxazole (1.25/23.75 µg) and piperacillin-tazobactam (30 –6 µg) disks [21]. Antibiotics were selected based on their frequent use in clinical practice and according to the EUCAST Clinical Breakpoint Tables v. 13.1 guideline for *Vibrio* spp.

### Molecular identification, whole genome sequencing and bioinformatics

Total genomic DNA (gDNA) was extracted using the Qiagen DNA Mini kit (Qiagen, Hilden, Germany), according to the instructions of the manufacturer. The Genesiq advanced real-time PCR kit (Genesiq, USA) was used for species identification targeting the outer membrane protein W (*omp*W). The *Vibrio cholerae* cholera enterotoxin subunit A gene *(ctx*A) was detected using an in-house quantitative polymerase chain reaction (qPCR) method [23]. In total, four *Vibrio cholerae* strains were subjected to WGS originating from clinical (*N* = 1, signed as Vibrio-33) and environmental (*N* = 3, signed as Vibrio-34, Vibrio-35, Vibrio-36) samples. Genomic libraries were prepared using the DNA prep Kit (Illumina, CA, United States) according to the instructions of the manufacturer. All isolates were paired-end sequenced using MiSeq Reagent Kit v2 with a read length of 2 × 150 base pairs on a MiSeq instrument (Illumina, CA, United States). Raw reads were de-novo assembled using the Ridom SeqSphere + software pipeline (v. 9.0.8, Ridom GmbH, Münster, Germany) and SKESA [24]. For the prediction of antimicrobial resistance (AMR) and virulence, draft genome sequences were analyzed using the Comprehensive Antibiotic Resistance Database (CARD) and the ResFinder database. In the SeqSphere + for reference sequences, the in-silico target scanning procedure was configured so that sequences were required to show at least 90% identity and 99% alignment to reference sequence [25–27]. Only resistance genes with a coverage of >80% and >75% identity were accepted. For virulence prediction and annotation, we used the Virulence Factor Database (VDFB) and rapid and standardized annotation of bacterial genomes with alignment-free sequence identification (Bakta) tool [26, 28]. For phylogenetic analysis and genomic visualization, multi locus sequence typing (MLST) data were extracted in silico from the whole genome sequencing (WGS) data according to the dedicated non-O, non-O139 *Vibrio cholerae* MLST scheme. Sequence types (STs) were determined using pubmlst database https://pubmlst.org/organisms/vibrio-cholerae [4]. Possible clonal relationships were investigated using core genome multilocus sequence typing (cgMLST) performed on Ridom SeqSphere + using the *Vibrio cholerae* scheme. To determine the cgMLST gene set, we performed a genome-wide gene-by-gene comparison using Ridom SeqSphere+. For comparison, 101 assembled NTVC genomes were downloaded from the National Center for Biotechnology Information (NCBI) genome database (Supplementary Material). Selection criteria included isolates from countries neighboring Hungary, and from those sharing a waterbody with Hungary.

### Literature review

In July 2024, a systematic literature review was conducted using Pubmed and Google Scholar without applying a time restriction. Additional articles were identified by checking the references of relevant articles, and duplicate articles were excluded. We included articles that described confirmed *V. cholera-*related infections in humans. Our search strategy included the following terms in Pubmed [ “non-O1 *Vibrio cholerae*“[MeSH Terms] OR non-O139 *Vibrio cholerae*“[MeSH Terms] OR “Non-toxigenic Vibrio” MeSH Terms] OR [ “non-O1 *Vibrio cholerae*“[TextWord] OR non-O139 *Vibrio cholerae*“[TextWord] OR “Non-toxigenic Vibrio” [TextWord] “non-O1, non-O139 *Vibrio cholerae* respiratory infection“[Text Word] OR “[TextWord] OR “*Vibrio Cholerae* pneumonia“[Text Word] AND “Vibrio Infections” [TextWord] OR “Vibrio Infections*“[Text Word] OR “Vibrio pneumonia*“[Text Word] OR “non-O1,non-O139 *Vibrio cholerae* pneumonia“[Text Word] in Google Scholar. In parallel, three authors screened the identified articles based on the defined parameters. We excluded non- *V. cholerae*, non-English articles, non- human infections, and environmental exposures. Clinical characteristics, risk factors, microbiological data, demographic data as well as treatment and clinical outcomes were collected and analyzed.

### Nucleotide sequence accession numbers

Sequences were deposited in GenBank under the accession number SUB13776351, BioProject PRJNA1007372.

## Results

### Microbiological results

#### Culture and biochemical identification methods and in vitro antimicrobial susceptibility

After 24 h, medium-sized yellow colonies appeared on TCBS agar, while BSA showed white medium-sized colonies with pronounced β-hemolytic zone. Purple colonies were observed on Vibrio ChromoSelect Agar. These were identified as *Vibrio cholerae* with high scores (> 2.3) using MALDI-TOF MS. Basic biochemical tests, including oxidase, catalase and indole tests, were performed with positive results. The slide-agglutination test identified the isolates as non-O1, non-O139 serogroups. The colonies from the concentrated water samples were not completely identical in appearance; therefore, three different colonies were selected for further analysis, including WGS, and all colonies were identified as *V. cholerae* by MALDI-TOF MS. The *V. cholerae* colony count of the surface water was 1300 CFU/100 mL. The clinical and all environmental isolates were susceptible to ceftazidime, cefotaxime, pefloxacin, doxycycline, meropenem, trimethoprim-sulfamethoxazole, piperacillin-tazobactam and erythromycin.

#### Whole genome sequencing

The average coverage was 99% (with an average depth of 84-fold); the N50 values were 205,412, 205,410, 178,740 and 178,754, respectively; and the average assembly size was 3.9 Mb with an average 47.5% GC content. A total of 3,481 coding sequences (CDS) were predicted in average. No putative plasmids were determined from raw data.

#### Detection of antimicrobial resistance genes (AMR), virulence genes and genomic comparison

All four isolates carried the *bla*_CARB–7_ and the *alm*EFG genes. Main virulence features were determined using VDFB. Common NTVC virulence genes were present, including VCA0109, *clp*B/*vas*G, *hly*-A, *icm*F/*vas*K, *lux*S, *rtx*B-D, *vas*A-L, *vip*A/*mgl*A, *vip*B/*mgl*B, *vgr*B-3 and *tox*R. Virulence profiles were identical between the sequenced clinical and environmental isolates. Some parts of the Vibrio 7th pandemic islands, VSP1 (VC0174 and VC0186) and VSP2 (VC0489, VC0495, VC0496, VC0497, VC0501a, VC0504, VC0505, VC0506, VC0507, VC0508, VC0509, VC0510, VC0517) were detectable, most of which without any known function. Clinical and environmental isolates were negative for cholera toxin (*ctx*A and *ctx*B), zonula occludens toxin (*zot*), accessory cholera enterotoxin *(ace*), toxin coregulated pilus (TCP) and heat-labile enterotoxin gene (*stn*) and the Type 3 secretion system (T3SS). The cgMLST results showed that the allelic distance between the clinical and three environmental isolates was zero, indicating that the isolates were identical. The in-silico analysis of MLST resulted in a novel combination of existing allele variants that were assigned as ST1605. Genomic comparison of isolates selected from NCBI and our isolates showed high allelic distance (20<), but we did not identify any possible epidemiological link.

#### Review results

**Fig. 1:**
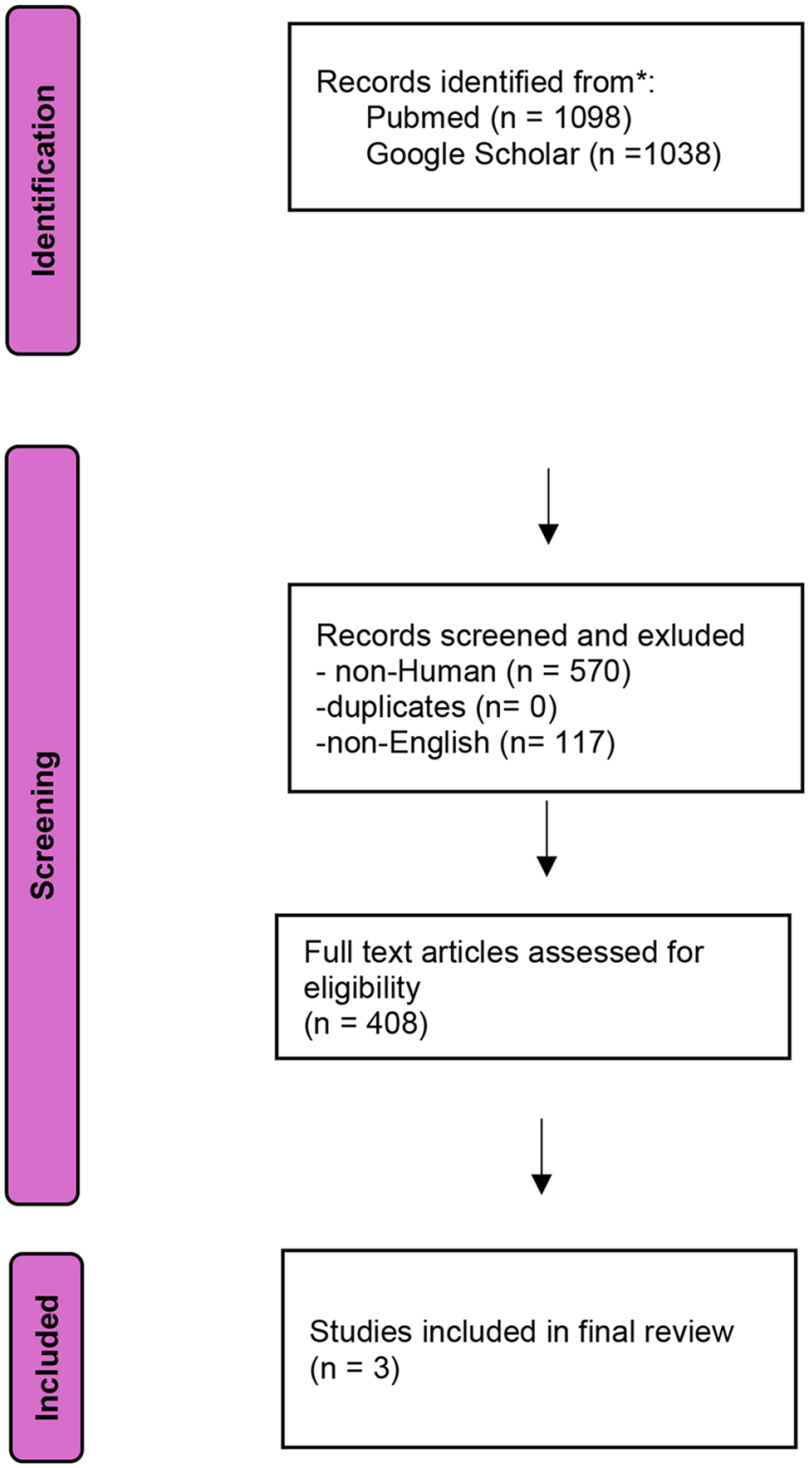
PRISMA flow diagram for selections of non-O1, non-O139 *Vibrio cholerae* infections

The initial search yielded 1,098 articles in PubMed and 1,038 articles in Google Scholar. We excluded 117 non-English and 570 non-human articles. The remaining articles were screened by title or abstract, 687 of them were excluded as they involved epidemiological studies, environmental or non-*V. cholerae* infection. After removing duplicate cases and including the present case, we identified a total of 4 adult (≥ 18 years of age) patients (3 males, 1 female) with non-O1, non-O139 *V. cholerae* related pulmonary infection (Table 1). The results were consistent with the three independent data collectors. A summary of the screening process is shown in the PRISMA flow diagram [29] (Fig. 1).

**Table 1:** Summary of cases described in literature of non-O, non-O139 Vibrio cholerae-related infections (including the present study)

Case ID	Year	Country	Demography	Source of infection	co-morbidity	Symptoms	Diagnosis	Specimen	Antimicrobials	Outcome	Reference
1.	2016	Italy	male,83 year old	unknown	COPD, alcohol abuse	Fever, tachycardia, shortness of breath, cough without production of sputum, mild jaundice, and abdominal pain.	Bacteraemia and secondary pneumonia caused by non-O1 non-O139 *V. cholerae*	Blood, stool, urine	iv. ceftriaxone for 2 weeks and a short course of 500 mg azithromycin	Discharged	[18]
2.	2006	New Mexico	male,67 year old	aspiration of water from the irrigation canal fed by a tributary of the Pecos River in Carlsbad, New Mexico, freshwater	alcoholism, tobacco use, and psoriasis	Fever, generalized weakness, cough, tachycardia	Sepsis and Pneumonia caused by non-O1 non-O139 V.cholerae	sputum, blood culture	imipenem/cilastin	Discharged	[17]
3.	2000	New Mexico	female, 25 year old	respiratory distress due to third degree burn	third degree burn	Fever, pneumonia	Sepsis and pneumonia caused by non-O1 non-O139 V. cholerae	sputum, blood culture	Empirical antibiotics, including cefazolin, aztreonam, and gentamicin	Died	[17]
4.	2023	Hungary	male,21 year old	aspiration of water from a lake designated for bathing in Central Hungary	none	Fever, shortness of breath, pneumonia	Primary pneumonia	Bronchoalveolar lavage	Amoxicillin-clavulanic acid, tienam, amphotericinB	Discharged	this study

#### Phylogenetic analysis

**Fig. 2:**
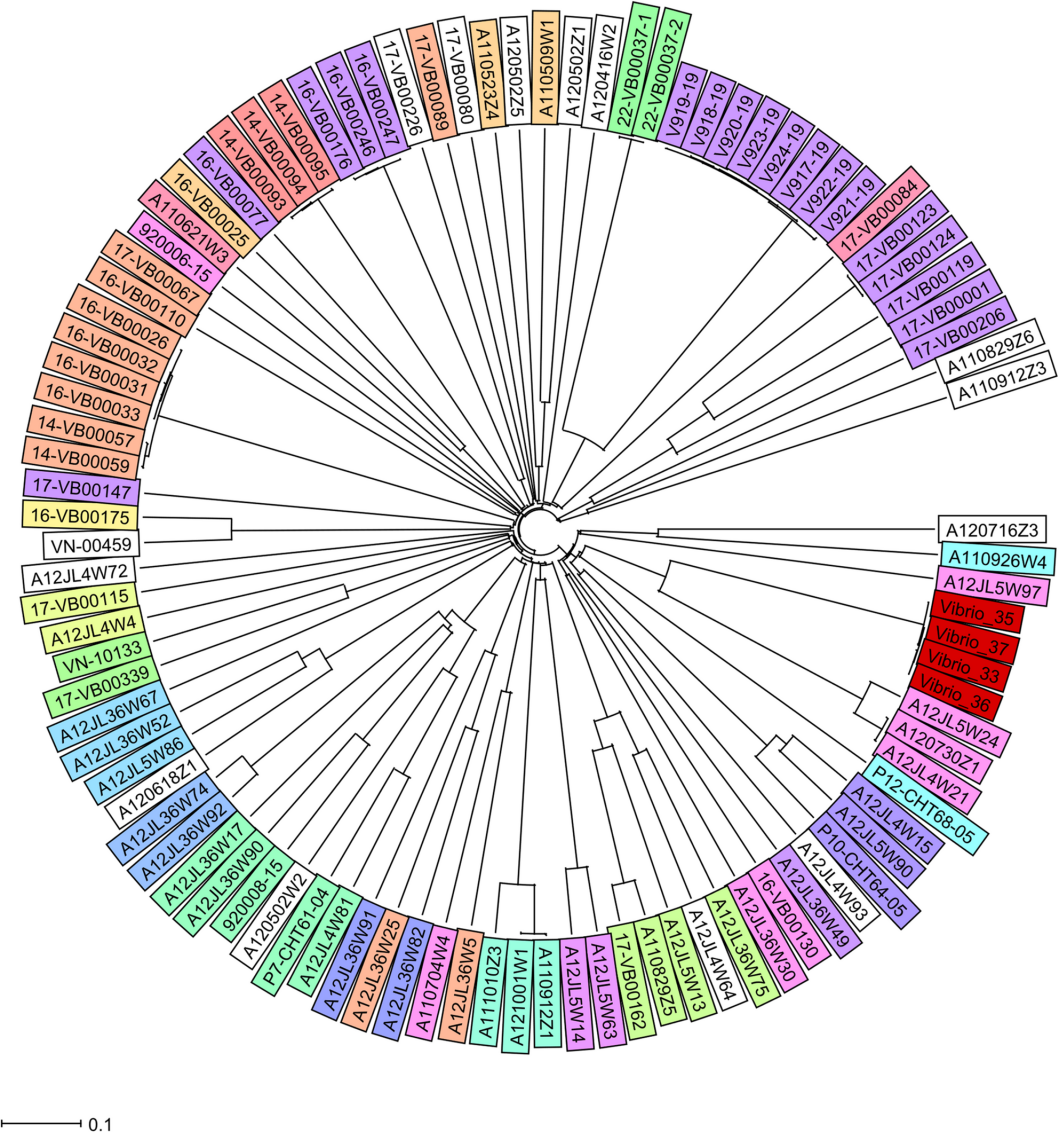
Neighbor-Joining Tree of the NTVC *Vibrio cholerae* based on 97 isolates. The data analysed by Ridom Seqsphere+ where missing pairwise values were ignored. The tree based on cgMLST NTVC *Vibrio cholerae* 2415 target with a cluster threshold ≤ 10. Different colors indicate sequence types. The isolate from this study is highlighted with red color. Each isolate is indicated in the supplementary

As of 2024 July, the pubMLST database contained 576 records from Europe, 209 of which represented isolates from human infections caused by non-O1, non-O139 serogroup; none of them were associated with pneumonia. All four isolates had the same MLST profile and represented a new ST classified as ST1605. The allele profile was the following: *adh* 1; *gyr*B 41; *mdh* 7; *met*E 273; *pnt*A 58; *pur*M 1; and *pyr*C 176. The cgMSLT analysis showed that the four isolates tested had identical cgMLST profiles and belonged to the same Cluster Type. In addition, we performed a core-genome-based phylogenetic analysis on the four isolates tested and an additional 101 selected isolates downloaded from the NCBI database (Supplementary Data). The relatedness of the isolates included in the analysis is shown in the phylogenetic trees generated from the CT analysis performed with Ridom Seqsphere+ (Fig. 2). The Vibrio-33 clinical sample showed 0 allele difference compared to the environmental isolates Vibrio-35, Vibrio-36 and Vibrio-37. Large allele distances were observed between the other selected isolates.

## Discussion

Pneumonia is an extremely rare and atypical manifestation of non-O1/non-O139 *Vibrio cholerae* infections [18, 30] we presented a case of an autochthonous pulmonary infection caused by non-O1, non-O139 *Vibrio cholerae* in Hungary after a near-drowning accident. By using WGS, we found that the clinical and environmental isolates were identical. The literature highlights the emergence of antibiotic resistance, in particular beta-lactam resistance, among NTVC isolates worldwide, which may have significant implications for national control strategies [5, 31]. Ampicillin or amoxicillin-clavulanate are the first-choice empiric antibiotics used in many countries, including Hungary, for the treatment of otitis, sinusitis or pneumonia, which are also the most common types of NTVC infections [19, 20, 32]. However, EUCAST has not established breakpoints for ampicillin and amoxicillin-clavulanate. Therefore, close partnership and ongoing communication between the microbiological laboratory and clinicians are essential to ensure appropriate antibiotic therapy in all NTVC cases.

Previously reported cases showed that pneumonia associated with near drowning is commonly caused by Gram-negative bacteria, especially *Aeromonas* sp. Therefore, if the clinician chose to use empiric antimicrobial therapy, in particular amoxicillin-clavulanate, they should always ensure coverage for these pathogens due to their potential for antibiotic resistance [5, 18, 32–34]. All four isolates carried the *bla*_CARB–7_ gene, which is a major determinant of beta-lactam resistance and common among *Vibrio cholerae* isolates [5]. Both isolates carried the *alm*EFG operon, which is a determinant antibiotic resistance to cationic antimicrobial peptides, including polymyxins. Polymyxins are widely used as a last-line treatment against MDR Gram-negative pathogens, potentially in cases of MDR NTVC infections. The main global regulator of the catabolite-sensitive operon was also detected, which, according to the literature, can also significantly increase resistance to oxacillin, azithromycin, erythromycin and crystal violet [35]. Among the isolates, NTVC-associated virulence genes were detectable using VDFB and Bakta. All isolates possessed RTX toxin, one of the most important virulence factors in NTVC isolates, which plays an important role in cellular rounding and depolymerization of the actin cytoskeleton in host cells. They also harbored hemolysin A (*hly*A), responsible for red blood cell lysis, and finally the type IV pilus, mannose-sensitive hemagglutinin (*msh*A), which is responsible for biofilm formation. Both isolates carried the *tox*R transcriptional regulator, which is one of the main and ancestral key virulence factors of vibrios, as well as *lux*R, a part of the quorum sensing system. Proteins associated with Type 6 Secretion System (T6SS)–*vas*A-L, *vip*A, *vib*B, vgrG-3, VC0109 were present [5, 36]. The allelic distance between the clinical and three environmental isolates was zero, indicating that the isolates were identical. In silico analysis of MLST resulted in a novel combination of existing allele variants, designated as ST1605. Genomic comparison of the selected isolates (*N* = 97) revealed no allelic differences between our isolates (*N* = 4), whereas a significant allelic distance (greater than 20) was observed in comparison with the other isolates. No additional genetic relationships were detected among the isolates tested.

Limited data are available on aspiration-associated pneumonia. However, organisms acquired through direct aspiration of water may result in serious bacterial infection due to opportunistic pathogens, including *Aeromonas* sp., *Staphylococcus aureus*, *Haemophilus influenzae* or *Pseudomonas aeruginosa* [37, 38]. but *Vibrio cholerae* is very rare. However, clarifying the etiology of a severe pulmonary status is not simple, as drowning can directly cause aspiration pneumonia, because water entering the lungs triggers anti-inflammatory reaction with the release of different pro-inflammatory cytokines [32]. We found only three cases in the literature where non-O1, non-O139 *Vibrio cholerae* caused freshwater-associated pneumonia. Two of these cases initially presented with bacteremia and pneumonia, which later developed complications, whereas only one case involved a primary pulmonary infection following a near-drowning event in the United States [18, 30]. To the best of our knowledge, this is the first reported case of primary pulmonary infection caused by *V. cholerae*, associated with a near-drowning event in Europe. Other vibrios, such as *Vibrio vulnificus* and *Vibrio metschnikovii*, can sometimes cause pneumonia directly, but specific virulence features involved in the etiology are unknown. All sequenced isolates showed the same virulence profile. None of the isolates carried significant virulence features, such as T3SS or VP-s, and no other specific toxins were found. Some parts of VSP1 and VSP2 were present; however, it is still unknown how they can contribute to virulence in humans [39].

As of July 2024, the presented case is the only identified case of NTVC infection at this bathing site, and the overall number of reported NTVC cases in relation to recreational bathing is very low (1–2 cases/year) in Hungary. Mild or asymptomatic cases, however, may go unnoticed. NTVC counts observed in this study were similar to those detected in other temperate lakes used for bathing, with or without recognized human NTVC infections [40, 41]. Despite the low incidence, severe NTVC infections may be a real health risk, especially with the observed increase in case numbers in Europe [42]. Surface waters, especially shallow lakes, are expected to become warmer and more saline due to climate change, leading to a higher prevalence of non-cholera *Vibrios* species. Subsequently, the number of NTVC cases is also expected to increase in temperate regions, potentially including severe, life-threatening infections as reported in the present study [41]. There are no established measures to control the proliferation of NTVC in surface water. Current guidelines recommend raising public awareness of the associated health risks and informing physicians about the potential for water-related vibriosis cases [43].

## Supplementary Information

Below is the link to the electronic supplementary material.

Supplementary Material 1

Supplementary Material 2

## Data Availability

Sequences have been deposited at the GenBank under the accession number SUB13776351, BioProject PRJNA1007372.

## Abbrevations

Ace: Accessory cholera enterotoxin
AMR: Antimicrobial resistance
ARDS: Adult respiratory distress syndrome
BAL: Bronchoalveolar lavage
CARD: The Comprehensive Antibiotic Resistance Database
CFU: Colony-forming unit
CDS: Coding sequences
ctxA: Cholera enterotoxin subunit A gene
ctxB: Cholera enterotoxin subunit B gene
CT: Cluster Type
cgMLST: Core genome multilocus sequence typing
ECDC: European Centre for Disease Prevention and Control
EUCAST: The European Committee on Antimicrobial Susceptibility Testing
FSA: Bovine serum albumin
ICU: Intensive Care Unit
MALDI TOF MS: Matrix-assisted laser desorption ionization time-of-flight mass spectrometry
MDR: Multidrug resistance
MLST: Multilocus sequence typing
NTVC: Non-toxigenic Vibrio cholerae
ompW: Outer membrane protein W gene
PCR: Polymerase chain reaction
qPCR: Quantitative polymerase chain reaction
PEEP: Positive end-expiratory pressure
SBA: Sheep blood agar
ST: Sequence Type
stn: Heat-labile enterotoxin
TCBS: Thiosulfate-citrate-bile salts-sucrose agar
TCP: Toxin gene toxin-coregulated pilus
T3SS: Type 3 secretion system
T6SS: Type 6 secretion system
VBNC: Viable but not culturable form
VDFB: Virulence Factor Database
VP: Vibrio pathogenic island
VSP1: Vibrio seventh pandemic island I
VSP2: Vibrio seventh pandemic island II
zot: Zonula occludens toxin
WGS: Whole genome sequencing
