# Supplementary material for: Genomic characterization of non-O1, non-O139 Vibrio cholerae causing rare clinical manifestation
Source: BMC Infect Dis. 2025 Dec 9;26:58. doi: 10.1186/s12879-025-12191-9 (PMC12801787; doi:10.1186/s12879-025-12191-9)
Supplement: Supplementary file 2 — Supplementary Material 2 [file 12879_2025_12191_MOESM2_ESM.pdf]

CARE Checklist (2013) of information to include when writing a case report

(cc) BY-NC-ND

Topic	Item	Checklist item description	Reported on Page
Title	1	The words "case report" should be in the title along with the area of focus	No
Key Words	2	2 to 5 key words that identify areas covered in this case report	14
Abstract	3a	Introduction—What is unique about this case? What does it add to the medical literature?	18
	3b	The main symptoms of the patient and the important clinical findings	18-21
	3c	The main diagnoses, therapeutics interventions, and outcomes	NA
	3d	Conclusion—What are the main "take-away" lessons from this case?	34-37
Introduction	4	One or two paragraphs summarizing why this case is unique with references	NA
Patient Information	5a	Demographic information and other patient specific information	NA
	5b	Main concerns and symptoms of the patient	66-80
	5c	Medical, family, and psychosocial history including relevant genetic information (also see timeline).	66-80
	5d	Relevant past interventions and their outcomes	66-80
Clinical Findings	6	Describe the relevant physical examination (PE) and other significant clinical findings	66-80
Timeline	7	Important information from the patient's history organized as a timeline	NA
Diagnostic Assessment	8a	Diagnostic methods (such as PE, laboratory testing, imaging, surveys)	3, 4, 5
	8b	Diagnostic challenges (such as access, financial, or cultural)	N
	8c	Diagnostic reasoning including other diagnoses considered	NA
	8d	Prognostic characteristics (such as staging in oncology) where applicable	NA
	9a	Types of intervention (such as pharmacologic, surgical, preventive, self-care)	NA
Therapeutic Intervention	9b	Administration of intervention (such as dosage, strength, duration)	77-78
	9c	Changes in intervention (with rationale)	NA
	10a	Clinician and patient-assessed outcomes (when appropriate)	NA
	10b	Important follow-up diagnostic and other test results	NA
Follow-up and Outcomes	10c	Intervention adherence and tolerability (How was this assessed?)	NA
	10d	Adverse and unanticipated events	NA

**Discussion**	**11a**	Discussion of the strengths and limitations in your approach to this case	NA
	**11b**	Discussion of the relevant medical literature	178-222
	**11c**	The rationale for conclusions (including assessment of possible causes)	178-22
	**11d**	The primary "take-away" lessons of this case report	214-222
**Patient Perspective**	**12**	When appropriate the patient should share their perspective on the treatments they received	NA
**Informed Consent**	**13**	Did the patient give informed consent? Please provide if requested	**Yes**
